# How reading behavior shapes physical and mental health: evidence from China

**DOI:** 10.3389/fpubh.2026.1876543

**Published:** 2026-07-08

**Authors:** Jiajia Li, Hanjing Zhang

**Affiliations:** Department of Economics and Management, Shanxi University, Taiyuan, China

**Keywords:** Healthy China strategy, heterogeneity, mechanisms, physical and mental health, reading behavior

## Abstract

Against the backdrop of the deepening development of nationwide reading and the continuous advancement of the Healthy China strategy, reading is not only an important direction for expanding public cultural services but also a crucial support for building a Healthy China. This paper utilizes data from the Chinese General Social Survey and employs ordered Probit regression models to systematically examine the effects and underlying mechanisms of reading behavior regarding residents’ physical and mental health. Findings indicate that reading behavior is significantly positively correlated with residents’ physical and mental health, with heterogeneity observed across urban–rural, regional, and age dimensions. Specifically, the health benefits are significantly greater for rural residents and those in central and western regions compared to urban residents and those in eastern regions. Furthermore, reading exerts a significant positive effect on the health of middle-aged and older adults, while exhibiting a suppressive effect among the young population. Further mechanism analysis reveals that reading improves residents’ physical and mental health through three pathways: enhancing self-cognition, improving subjective social status, and reducing health risk perception bias. Therefore, governments should continuously optimize public cultural services and implement differentiated reading promotion strategies targeting different regions and age groups, so as to maximize the health benefits of reading and provide strong support for improving residents’ health and advancing the Healthy China strategy.

## 1 Introduction

Health serves as a cornerstone of both high-quality economic growth and individual well-being—a fundamental determinant that not only underpins the pursuit of a better life but also constitutes a vital pillar of sustainable social development. In recent years, China has witnessed a steady increase in average life expectancy, alongside notable progress in the Healthy China initiative. Nevertheless, despite sustained economic growth and policy advancements, the health status of Chinese residents continues to face formidable challenges. The accelerating pace of population aging, coupled with escalating mental stress, has contributed to a rising prevalence of chronic diseases, while mental disorders are increasingly affecting younger populations. As a result of these trends, the population experiences a decline in both quality of life and subjective well-being. Meanwhile, the public health system faces increasingly heavy burdens. In this context, the continued advancement of nationwide reading initiatives presents a significant opportunity to improve public health. In 2024, the comprehensive reading rate among Chinese adults—encompassing both print and digital publications—reached 82.1%, while the book reading rate stood at 59.9%. Reading has increasingly become a widely adopted social and cultural practice. Moreover, the multifaceted value of reading is gaining scholarly attention, as it not only facilitates the acquisition of knowledge and information but may also exert positive effects on physical and mental health through mechanisms such as cognitive restructuring and emotional regulation. In the context of the 20th CPC National Congress, health was identified as a vital indicator of national prosperity and strength. Building on this, the 15th Five-Year Plan has further elevated health to the level of a national strategic priority. Against the backdrop of comprehensively advancing the Healthy China initiative, the exploration of diverse health promotion strategies holds substantial practical significance. Nevertheless, scholarly attention to the nexus between reading and health remains limited. Existing research has largely focused on the therapeutic value of reading, with few studies systematically investigating the health implications of reading behavior or the underlying mechanisms that mediate such effects. To address this gap, this paper utilizes data from the 2021–2023 multiple waves of the China General Social Survey (CGSS). It aims to clarify the effects, heterogeneity, and mechanisms through which reading behavior influences residents’ health, thereby providing a theoretical foundation for advancing nationwide reading initiatives and the Healthy China initiative.

## 2 Literature review

Existing research on physical and mental health has primarily focused on two key dimensions: measurement methodologies and determinants. Regarding measurement, no consensus has been reached on a unified standard, as health is defined not merely by the absence of disease but by a holistic state of well-being encompassing physical, mental, and social dimensions. In contemporary academia, health measurement is largely approached from both macro and micro perspectives, with the selection of appropriate indicator systems depending on research objectives, data quality, and specific contexts. At the macro level, health is commonly assessed using health-related input factors (1). Previous studies have employed life expectancy and mortality rates as indicators of national health status (3), whereas others have adopted the logarithm of annual total medical expenditure as a proxy measure, which exhibits a negative association with the health of older adults (4). At the micro level, self-rated health status has emerged as the predominant indicator for assessing individual health. Although self-rated scores are inherently subjective, this measure is readily accessible and demonstrates strong correlations with other objective health indicators (5), thereby serving as a reliable reflection of individual health to a considerable extent. Drawing on data from the China Family Panel Studies, existing research has incorporated self-rated health as a key component of broader health indicators (6). Advancing this line of inquiry, subsequent studies have further disaggregated health into four dimensions—self-rated health, depression risk, social adaptation, and well-being—and applied principal component analysis to construct a more comprehensive health index, with higher index values corresponding to better health outcomes (7).

Beyond measurement approaches, a substantial body of literature has investigated the determinants of physical and mental health. Given that health is shaped by the interplay of multiple factors, its determinants are correspondingly diverse. Existing research has largely examined these factors from both macro and micro perspectives. At the macro level, institutions, policies, and the environment constitute the fundamental social context that shapes the developmental trajectory of health. The influence of these factors is primarily reflected in three dimensions. First, with respect to the socioeconomic environment, a conducive economic growth environment has been shown to increase health demand and health expenditure among residents, thereby contributing to improved health outcomes (8). Second, concerning public infrastructure, studies examining the rural toilet renovation program have demonstrated that in areas with limited healthcare accessibility, the implementation of sanitation infrastructure—such as improved sanitary toilets—can effectively reduce disease transmission risks and yield significant improvements in resident health (1). Third, with regard to healthcare security policies, research employing cost–benefit analysis indicates that in regions characterized by substantial individual medical burdens, the expansion of commercial health insurance enhances medical service utilization, alleviates health risks, and ultimately contributes to better health outcomes. This positive effect is particularly pronounced for long-term health insurance products (10). Moreover, well-established healthcare policies have been shown to exert a positive influence on resident health (11). However, some scholars contend that macro-level factors may not necessarily yield positive health outcomes; rather, they may generate adverse effects through mechanisms such as environmental degradation. In this regard, Environmental regulation has been demonstrated to effectively mitigate the detrimental impact of environmental pollution on residents’ physical and mental well-being (12).

From a micro-level perspective, both personal attributes and lifestyle behaviors exert a direct influence on health outcomes. Individual-level determinants of physical and mental health encompass gender, marital status, educational attainment, income, and other sociodemographic characteristics. Regarding marital status, the protective role of marriage in individual health has been empirically well established. Research has shown that, compared with their rural counterparts, urban women’s health is more significantly affected by spousal influence (13). Moreover, the health-protective effect of marriage is more pronounced among individuals with higher socioeconomic status (14). With respect to lifestyle behaviors, positive practices—such as regular physical exercise—have been shown to enhance health status to a certain extent, whereas adverse behaviors, including smoking and excessive alcohol consumption, directly impair physical health (15, 67). Despite the richness of existing research on resident health, the cultural dimension has received scant attention. In particular, few scholars have investigated the underlying causes of health disparities among residents through the lens of reading behavior.

The relationship between reading behavior and physical and mental health has long been a subject of scholarly inquiry. Its intellectual origins can be traced to ancient Greece, where books were regarded as therapeutic instruments. By the 19th century, the therapeutic value of reading had become primarily associated with mental health. It was not until the 1980s, following its introduction to China, that domestic scholars began systematic investigations into the intrinsic link between reading and health. The existing literature addresses three primary dimensions: influence effects, moderating factors, and population differences. First, regarding influence effects, bibliotherapy has received the most extensive scholarly attention. Existing studies consistently indicate that reading-based interventions are effective in treating depression, with efficacy comparable to that of conventional therapies (16). Subsequent research has further expanded this line of inquiry, revealing that reading activities not only alleviate psychological disorders such as anxiety and depression (17). First, through the acquisition and cognitive processing of health information, reading helps individuals form accurate disease perceptions and behavioral decisions (18–20). Second, through emotional resonance with narrative texts, reading facilitates emotional regulation and psychological counseling (21–23). Third, through the presentation of behavioral modeling in texts, reading triggers observational learning and behavioral imitation (24). Fourth, the positive emotional experience generated by the reading process itself. Behavioral health research indicates that health behaviors that can be sustained over the long term depend not only on individuals’ rational awareness of health benefits, but also on the immediate pleasure and intrinsic satisfaction derived from the activity itself (1, 2, 25). As a low-cost, self-directed cultural activity, reading provides exactly such intrinsic rewards—pleasure, emotional resonance, and sense of meaning—which continuously reinforce individuals’ behavioral engagement, thereby conferring a unique advantage in mental health promotion. Nevertheless, it is worth noting that some studies, from the perspective of information behavior, have indicated that inappropriate or excessive reading behaviors may pose potential risks to healthy individual development. For instance, addictive online novel reading may immerse readers in fantasy, potentially impairing daily functioning and social participation over the long term (26). Accordingly, attention should be directed toward guiding reading behaviors to better fulfill their positive role in promoting mental health. Second, regarding moderating factors that influence the health benefits of reading behavior, existing studies have shown that reading medium (9), type of reading content (27–30), and individual reading motivation (31) all affect the intensity and direction of reading’s impact on health. Finally, regarding population differences, the academic community has focused on health disparities associated with reading across different populations. For instance, among the older adult, the role transition following retirement and the health challenges associated with aging render this population more susceptible to mental health issues. Reading, however, helps fulfill their core needs, such as emotional connection and self-improvement (32), thereby not only promoting mental health but also serving as a preventive measure against potential health problems. Beyond the older adults, research has also confirmed the positive effects of reading on physical rehabilitation in patients with chronic diseases (33) and on anxiety relief among college students (34, 35). In recent years, research focusing on children has further indicated that reading habits not only promote language and cognitive development but also help address tension and fear through materials such as picture books, thereby fostering positive psychological traits (36).

In summary, existing research on the determinants of residents’ physical and mental health has predominantly adopted macro-level social and micro-level individual perspectives. However, limited attention has been paid to the cultural dimension, with only a handful of studies establishing a link between reading behavior and health outcomes. Moreover, when examining this relationship, most studies have concentrated on the therapeutic value of reading as an intervention, whereas research utilizing micro-level data to systematically investigate its effects and underlying mechanisms remains scarce. In light of this, the present study draws on individual-level microdata to explore the direct effects and indirect mechanisms of reading behavior on residents’ physical and mental health. Focusing on two core dimensions—physical health and mental health—this research is situated within the context of China’s National Reading Initiative and the Healthy China strategy. Furthermore, it incorporates urban–rural disparities, regional imbalances, and age differences into the analytical framework to compare the heterogeneous effects of reading on health across diverse populations. Accordingly, this study seeks to offer a cultural perspective, providing both theoretical insights and empirical evidence to inform public health promotion efforts.

The potential marginal contributions of this study are as follows. First, from the perspective of research orientation, unlike existing studies that examine the determinants of health from macro-level environmental and micro-level individual characteristics, this study identifies reading behavior as the primary cultural practice variable. Drawing on individual-level microdata, it employs self-reported physical and mental health indicators to capture health outcomes across multiple dimensions and empirically examines both the effects and underlying mechanisms of reading behavior. Accordingly, it constitutes a valuable addition to existing research on the determinants of health and offers a novel perspective on health improvement from a cultural standpoint. Second, with respect to research methodology and mechanisms of influence, this study leverages rich and detailed microdata to further elucidate, across multiple dimensions, the mechanisms through which reading behavior affects residents’ physical and mental health—specifically, via enhanced cognitive ability, improved subjective social status, and reduced health risk perception bias. Compared with existing studies, this study provides a clear explanation, from a cultural perspective, of the direct effects and indirect pathways through which reading behavior influences residents’ physical and mental health, thereby addressing the gap in the empirical examination of the underlying mechanisms in prior research. Finally, in terms of research content, this study systematically examines the differential effects of reading behavior on health across diverse populations, including different regions and age groups. The findings reveal that, within the context of uneven development, the health-promoting effects of reading are more pronounced among rural residents, residents in central and western China, and middle-aged and older adults. These results provide a theoretical foundation for implementing targeted and differentiated health interventions. As well as micro-level evidence for public cultural institutions—particularly libraries—in developing reading-based health promotion services.

## 3 Theoretical foundations and research hypotheses

### 3.1 Reading behavior and physical–mental health

Social cognitive theory, proposed by American psychologist Bandura, posits that individual activity is driven by multiple interrelated factors, namely environment, individual cognition, and behavior (37). A central tenet of this theory is the reciprocal interaction between individual cognition and the social environment, suggesting that behavior is shaped by both subjective personal factors and contextual influences (38). Individual factors primarily include cognitive level, self-efficacy, and social comparison, whereas environmental factors encompass external role models, feedback, and opportunities for self-evaluation (39). On the one hand, reading serves as a vital channel for acquiring information and knowledge, systematically enhancing individuals’ health-related cognition and self-efficacy. Through the acquisition of health knowledge, skills, and practices via reading, individuals not only deepen their understanding and judgment of health issues but also strengthen their psychological confidence, thereby facilitating health-promoting behaviors. On the other hand, the media constitute a critical avenue for observational learning and the internalization of social norms, with reading—particularly of books and newspapers—representing a specific form of media engagement. By engaging with the characters and narratives constructed in media, readers encounter potential “external role models” for observational learning. Observing the behavioral patterns of these characters, such as healthy behaviors and emotion regulation strategies, provides individuals with accessible models to emulate in their pursuit of physical and mental well-being.

Furthermore, from the perspective of sedentary behavior types, reading can be regarded as a cognitively active sedentary behavior. Studies suggest that sedentary behavior should not be treated as homogeneous, as the effects of cognitively active sedentary behavior on brain and mental health may differ from those of cognitively passive behavior (68). Although reading is often performed while sitting, it involves sustained attention, semantic processing, imagination, and knowledge acquisition, which distinguishes it from cognitively passive sedentary activities and makes it more likely to exert positive effects on health.

Given China’s large population, residents exhibit significant heterogeneity across urban–rural, regional, and age dimensions. These differences manifest not only in daily behaviors—such as reading habits and health knowledge—but also in more fundamental aspects, including access to social resources. Consequently, they influence the extent of residents’ engagement with reading and shape the varying outcomes observed in their physical and mental health development. Specifically, across age groups, variations in social environment, leisure time, and learning capacity lead to distinct reading behaviors among middle-aged and young adults compared with older adults, which in turn result in differential effects on physical and mental health. From an urban–rural perspective, systemic differences in governance models, socioeconomic conditions, and public cultural infrastructure lead to marked disparities in the knowledge systems, social resources, and learning environments accessible to residents. These disparities influence reading frequency and ultimately contribute to differentiated effects on physical and mental health. From a regional perspective (eastern, central, and western), variations in economic development, lifestyle, and investment in cultural education result in disparities in educational resources and leisure time available to residents. Consequently, the health benefits of reading behavior may be largely constrained by regional differences in resource accessibility and reading opportunities. Based on the above analysis, we propose the following hypothesis:

*Hypothesis 1:* Reading behavior is positively correlated with residents’ physical and mental health, with heterogeneity observed across urban–rural, regional, and age dimensions.

### 3.2 Mechanisms of reading on physical and mental health

Firstly, reading promotes residents’ physical and mental health through individual cognitive ability. Cognitive ability refers to the capacity of the human brain to perform a series of information processing operations, including encoding, storage, and retrieval (40). This capacity for efficient information processing provides individuals with a solid foundation for deeply understanding phenomena and grasping underlying patterns. Cognitively active leisure activities can protect cognitive function and brain health throughout the human lifespan (68). As a typical cognitively active leisure activity, reading helps people make more rational health-related decisions by accumulating cognitive reserve and optimizing individuals’ ability to process health information.

Empirical evidence indicates that the cognitive patterns readers employ when interpreting fictional plots and character behaviors in literary works—such as novels and biographies—directly shape their understanding of the real world (41). Furthermore, by engaging with books and materials across diverse fields, readers construct their own knowledge and comprehension—a process that cultivates and enhances their cognitive abilities. Enhanced cognitive ability, in turn, affects physical and mental health. On one hand, higher cognitive ability facilitates the understanding of health patterns and enables a deeper comprehension of how various factors—such as physiological aging, lifestyle habits, and disease—shape changes in health status. It also strengthens health awareness and encourages proactive intervention. On the other hand, individuals with higher cognitive ability not only value health but also translate this awareness into sustainable health behaviors. Research has shown that cognitive ability enhances individuals’ willingness to engage in physical exercise (42, 43) and promotes the adoption of healthy lifestyles. Based on the above analysis, we propose the following hypothesis:

*Hypothesis 2a:* Reading behavior can enhance individual cognitive ability, thereby enabling residents to achieve better health outcomes.

Secondly, Reading influences residents’ physical and mental health through the enhancement of individuals’ subjective social status. Subjective social status is defined as an individual’s perception of their position within the social hierarchy (44). Reading has traditionally been seen as a key pathway to enhancing social standing. It not only helps individuals secure better jobs and faster promotions (45), thereby improving their objective social standing, but also facilitates access to extensive social networks and reduces social exclusion (46). These processes prompt individuals to reassess their own social standing, helping to reduce the sense of relative deprivation arising from social comparison, and thereby fostering a more positive and optimistic self-perception of their social status. An elevated subjective social status, in turn, influences residents’ physical and mental health. Since the 1990s, a growing body of literature has explored the relationship between subjective social status and health, yielding substantial progress in this area. A large body of research has demonstrated that, compared with objective indicators of social status—such as occupation and income—subjective social status exhibits a stronger association with health (47, 48) and serves as an important predictor of health outcomes. Specifically, lower subjective social status is associated with poorer health outcomes. This association is evident not only in physical conditions such as hypertension and diabetes (49) but also in psychological functioning and health-related factors, including body fat distribution, heart rate, and stress coping (47). Conversely, higher subjective social status encourages individuals to place greater value on their health and translate this awareness into positive health behaviors—such as regular exercise and a balanced diet—thereby improving physical health. In addition, it fosters a more optimistic outlook toward the future and alleviates negative emotions such as stress and anxiety, thereby contributing to mental health. Based on the above analysis, we propose the following hypothesis:

*Hypothesis 2b:* Reading behavior can enhance individuals’ subjective class perception, thereby enabling residents to achieve better health outcomes.

Thirdly, Reading promotes residents’ physical and mental health by alleviating cognitive bias toward health risks. Cognitive bias, which originated in cognitive psychology and was later incorporated into behavioral economics, generally refers to the tendency of individuals to rely on a limited set of heuristic principles to simplify complex tasks, thereby producing deviations from factual reality (50). Research has shown that certain groups exhibit inaccurate perceptions and judgments regarding their own health risks (51). Such inaccuracies often stem from variations in educational background, cognitive ability, and other individual characteristics, leading individuals to make judgments that deviate from factual reality when assessing health risks, and consequently to engage in decisions and behaviors detrimental to their health. As a core pathway for acquiring and comprehending health information, reading directly shapes individuals’ judgments regarding health threats. Specifically, reading helps individuals acquire scientific and systematic health knowledge and establish a more objective and comprehensive risk perception framework, thereby effectively correcting cognitive biases—such as excessive fear or unwarranted complacency—that may arise in health judgment contexts. Consequently, individuals are better equipped not only to make sound health decisions and engage in preventive health behaviors—thereby enhancing physical fitness—but also to reduce the psychological burden associated with unnecessary health anxiety, thus improving mental health. Based on the above analysis, we propose the following hypothesis:

*Hypothesis 2c:* Reading behavior can reduce health risk perception bias, thereby enabling residents to achieve better health outcomes.

## 4 Research design and methodology

### 4.1 Model specification

Given that the dependent variables, physical health and mental health, are both measured as ordinal discrete variables, the ordered Probit model is adopted for estimation:

$$\text{healt}h_{\mathrm{it}}=\alpha +\text{βrea}d_{\mathrm{it}}+\gamma X_{\mathrm{it}}+\delta_{t}+\varphi_{p}+\varepsilon_{\mathrm{it}}$$ (1)

where $\text{Physica}l_{\mathrm{it}}$ and $\text{Menta}l_{\mathrm{it}}$ represent the physical health and mental health of individual i in period t , respectively; $\mathrm{rea}d_{\mathrm{it}}$ denotes the reading frequency of individual i in period t ; $X_{\mathrm{it}}$ is a vector of control variables; $\mu_{t}$ and $\lambda_{p}$ indicate time fixed effects and province fixed effects, respectively; and $\varepsilon_{\mathrm{it}}$ is the random error term.

### 4.2 Variable selection

#### 4.2.1 Dependent variable

The dependent variable of this paper is overall health, which is operationalized through two dimensions—physical health and mental health—to comprehensively capture individuals’ health status. Following the approach of Deng and Feng (52), physical health is measured using respondents’ self-assessed answers to the question: “How would you rate your current physical health status?” The five ordinal response options—"very unhealthy,” “relatively unhealthy,” “fair,” “relatively healthy,” and “very healthy”—are coded as 1, 2, 3, 4, and 5, respectively, with higher scores indicating better physical health. Mental health is assessed based on respondents’ answers to the question: “Over the last month, how frequently have you experienced feelings of depression or sadness?” The five response options—"always,” “often,” “sometimes,” “rarely,” and “never”—are coded as 1, 2, 3, 4, and 5, respectively, with higher values reflecting better mental health.

#### 4.2.2 Core explanatory variable

The core independent variable of this paper is reading behavior. Drawing on the method of Wang et al. (2), we measure reading behavior by reading frequency, as reported by respondents’ answers to the following question: “How often do you engage in the following activities during your leisure time over the past year?” with specific reference to “reading books, newspapers, or magazines.” The five ordinal response categories—“never,” “once a year or less,” “several times a month,” “several times a week,” and “every day”—are coded as 1, 2, 3, 4, and 5, respectively, with higher scores indicating a greater frequency of reading.

#### 4.2.3 Mechanism variables

Cognitive ability is measured using questions from the individual cognitive module of the CGSS questionnaire, with response options ranging from “completely unable to understand,” “relatively poor,” “average,” “relatively good,” to “very good” (52).

Subjective class identity refers to an individual’s subjective perception of their own position within a social hierarchy. It is measured using the following question from the CGSS questionnaire: “Overall, where do you think you currently stand in society?” Respondents provide a self-rated score on an integer scale from 0 to 10, with higher scores indicating a higher subjective class identity (53).

Health risk perception bias is defined as the deviation between an individual’s subjective assessment of health threats and the objective state of those threats. Following the methodology of Han et al. (54) and considering data availability, this paper operationalizes health risk perception bias using only the 2021 data, as detailed below. (1) Subjective health status is measured using the question: “Overall, how would you rate your health status (including both physical and mental health)?” The five ordinal response options, ranging from “poor” to “very good,” are coded as 1 to 5, respectively, with higher scores indicating better subjective health. (2) Objective health status is assessed using the following questions: “During the past 4 weeks, how often have you experienced the following problems—physical pain, feeling unhappy or depressed, losing confidence in yourself, and feeling unable to overcome difficulties?” and “During the past 12 months, how often have you visited a traditional Chinese medicine practitioner or a Western medicine doctor?” The five ordinal response options, ranging from “never” to “very frequently,” are coded as 1 to 5, respectively, with higher scores reflecting poorer objective health status.(3) Health risk perception bias is then computed as the absolute value of the ratio of subjective health status to objective health status.

#### 4.2.4 Control variables

To mitigate bias arising from omitted variables, this paper introduces individual characteristics, household characteristics, and social characteristics as control variables, drawing on relevant studies (42, 55). Specifically, individual-level variables include gender, age, household registration, educational level, religious belief, and physical exercise. Household-level variables include total household income. Social-level variables include social fairness and social trust.

### 4.3 Data sources and descriptive statistics

Conducted by the National Survey Research Center at Renmin University of China, the Chinese General Social Survey (CGSS) provides nationally representative data for this study. The survey is implemented nationwide and includes 28 provincial-level administrative regions in mainland China, with the exception of Xinjiang, Tibet, Hainan, Hong Kong, Macao, and Taiwan. Using standardized questionnaires, it collects multidimensional social data, including community environment, household characteristics, and individual behaviors. This paper selects seven waves of survey data from 2012, 2013, 2015, 2017, 2018, 2021, and 2023. After excluding observations with missing values, refusals to answer, or “do not know” responses, the final sample comprises 62,880 valid observations.

Descriptive statistics for all variables are presented in Table 1. The mean value of reading behavior is 2.2068, indicating that the overall reading frequency of Chinese residents is below the medium level. The mean value of physical health is 3.5484, which is close to “relatively healthy” but still far from “very healthy.” The mean value of mental health is 3.8677, placing it at an upper-medium level. Regarding control variables, males account for 48.63% of the sample and females for 51.37%. The mean age of respondents is 50.51 years. Urban household registration accounts for 44.55%, while rural household registration accounts for 55.45%. The mean value of educational attainment is 2.4046, indicating that the average education level of respondents is above junior high school. The proportion of respondents engaging in physical exercise is 16.44%. Approximately 48.78% of residents perceive society as fair, and 64.50% hold a trusting attitude toward others. The mean value of total household income is 66,793 yuan.

**Table 1:** Descriptive statistics.

Variable name	Variable definition	Means	SD	Min	Max	*N*
Reading behavior	Reading frequency, coded from 1 (lowest) to 5 (highest)	2.2068	1.3921	1	5	62,880
Physical health	Self-rated physical health, coded from 1 (lowest) to 5 (highest)	3.5484	1.0906	1	5	62,880
Mental health	Self-rated mental health, coded from 1 (lowest) to 5 (highest)	3.8677	0.9925	1	5	62,880
Gender	1 = male, 0 = female	0.4863	0.4998	0	1	62,880
Age	Survey year minus birth year (years)	50.5162	16.36	19	85	62,880
Household registration	1 = urban, 0 = rural	0.4455	0.497	0	1	62,880
Educational level	1 = primary school or below, 2 = junior high, 3 = senior high, 4 = college or above	2.4046	0. 9,210	1	4	62,880
Religious belief	1 = religious, 0 = non-religious	0.1062	0.3081	0	1	62,880
Exercise	1 = exercise, 0 = no exercise	0.1644	0.3706	0	1	62,880
Social fairness	1 = fair, 0 = unfair	0.4878	0.4999	0	1	62,880
Social trust	1 = trusting, 0 = not trusting	0.645	0.4785	0	1	62,880
Total household income	Total household income (10,000 RMB)	6.6793	8.0758	0	50	62,880

## 5 Empirical analysis

### 5.1 Baseline regression

This paper uses an ordered Probit model to assess how reading behavior affects residents’ physical and mental health. The baseline regression results are presented in Table 2. Columns (1) and (3) include only year and province fixed effects, while columns (2) and (4) further add control variables at the individual, household, and social levels. The regression results indicate that reading behaviors show a significant positive correlation with both physical health and mental health. Regarding physical health, this may be due to the nature of reading as an efficient means of information acquisition, compensating for deficiencies in health literacy and facilitating optimal health-related behaviors. Additionally, it serves as a systematic channel for acquiring scientific health knowledge, thereby cultivating awareness of self-monitoring and enhancing health literacy. Regarding mental health, the possible reasons are as follows. On the one hand, while reading literary works, readers can deeply experience the emotional world of the characters and generate emotional resonance, thereby enhancing their emotional regulation ability and improving mental health. On the other hand, reading books can help readers understand the mechanisms underlying the emergence and effects of psychological issues such as anxiety and depression, shift their perceptions of these conditions, and enhance their confidence in treatment, thereby alleviating psychological burden. Regarding the control variables, the estimated coefficients for education level, household registration status, family income, and physical exercise are significantly positive. This suggests that improvements in education level, acquisition of urban household registration, increases in family income, and greater frequency of physical exercise are associated with corresponding improvements in residents’ health status. In contrast, the estimated coefficients for age and religious belief are significantly negative, indicating that aging and adherence to religious beliefs are associated with a decline in residents’ physical and mental health.

**Table 2:** Baseline regression.

Variable	Physical health		Mental health	
	(1)	(2)	(3)	(4)
Reading behavior	0.1177^***^ (0.0033)	0.0441^***^ (0.0037)	0.0788^***^ (0.0033)	0.0231^***^ (0.0033)
Education level		0.0549^***^ (0.0066)		0.0422^***^ (0.0067)
Household registration		0.0355^***^ (0.0108)		0.0804^***^ (0.0109)
Gender		0.1318^***^ (0.0087)		0.1317^***^ (0.0088)
Age		−0.0265^***^ (0.0003)		−0.0066^***^ (0.0003)
Total household income		0.0097^***^ (0.0006)		0.0058^***^ (0.0006)
Religious belief		−0.0559^***^ (0.0147)		−0.0822^***^ (0.0148)
Physical exercise		0.1791^***^ (0.0121)		0.2430^***^ (0.0124)
Social fairness		0.1568^***^ (0.0090)		0.1963^***^ (0.0091)
Social trust		0.0813^***^ (0.0094)		0.1300^***^ (0.0094)
Year FE	Yes	Yes	Yes	Yes
Province FE	Yes	Yes	Yes	Yes
*N*	62,880	62,880	62,880	62,880

Robust standard errors are reported in parentheses. *, **, and *** indicate statistical significance at the 10, 5, and 1% levels, respectively.

### 5.2 Robustness checks

To enhance the reliability of the research findings, this study conducts robustness checks from two perspectives: (1) model substitution and (2) substitution of the core independent variable.

#### 5.2.1 Alternative model specifications

The physical and mental health data obtained from survey respondents are ordinal in nature and are typically analyzed using ordered Probit or ordered Logit models. However, empirical studies suggest that treating physical and mental health as continuous variables does not fundamentally alter the empirical results (56). Accordingly, we re-estimate the regressions using ordinary least squares (OLS) and ordered Logit models to assess the consistency of the findings. Results in Table 3 demonstrate consistently positive coefficients for reading behavior across all model specifications, thereby validating the robustness of the baseline findings.

**Table 3:** Robustness checks.

Variable	Logit		OLS		Replace core explanatory variable	
	Physical health	Mental health	Physical health	Mental health	Physical health	Mental health
Reading behavior	0.0748*** (−0.0063)	0.0367*** (−0.0064)	0.0418*** (−0.0034)	0.0215*** (−0.0033)	0.0551*** (−0.0044)	0.0408*** (−0.0045)
Control variables	Yes	Yes	Yes	Yes	Yes	Yes
Year FE	Yes	Yes	Yes	Yes	Yes	Yes
Province FE	Yes	Yes	Yes	Yes	Yes	Yes
*N*	62,880	62,880	62,880	62,880	62,996	62,996

*, **, and *** indicate statistical significance at the 10, 5, and 1% levels, respectively.

#### 5.2.2 Alternative measures of the core explanatory variable

Following the approach of Yin et al. (57), this study adopts respondents’ frequency of newspaper reading over the past year as a proxy variable and incorporates it into the regression model as a robustness check. Table 3 shows that when measured by newspaper reading frequency, reading behavior exerts a positive and statistically significant impact on residents’ physical and mental health at the 1% significance level. This result aligns with the baseline findings, thereby further reinforcing the credibility of the research conclusions.

### 5.3 Endogeneity treatment

Propensity score matching reduces estimation bias caused by sample selection by balancing observable covariates. Accordingly, the sample is divided into treatment and control groups based on reading frequency. Respondents reporting reading frequencies of “several times a month,” “several times a week,” or “every day” are assigned to the treatment group, while the remaining respondents constitute the control group. Using the control variables from the baseline model as matching covariates, both 1:1 nearest neighbor matching and radius matching are performed. After successfully passing the balance test, regression analysis is conducted on the matched sample. The results, presented in Table 4, show that the coefficients remain consistently positive, indicating that reading behavior has a significant beneficial impact on residents’ physical and mental health.

**Table 4:** Endogeneity treatment.

Variable	Nearest neighbor matching		Radius matching	
	Physical health	Mental health	Physical health	Mental health
Reading Behavior	0.1215^***^ (0.0361)	0.2695^**^ (0.0181)	0.0720^***^ (0.0207)	0.1654^***^ (0.0181)
Control Variables	YES	YES	YES	YES
Year FE	YES	YES	YES	YES
Province FE	YES	YES	YES	YES
N	62,876	62,876	62,879	62,879

*, **, and *** indicate statistical significance at the 10, 5, and 1% levels, respectively.

### 5.4 Heterogeneity analysis

Due to the persistent challenges of unbalanced and inadequate development in contemporary Chinese society, notable disparities exist across different population groups, primarily reflected in the urban–rural binary structure, regional development levels, and age cohort characteristics. Accordingly, this paper further examines how the effect of reading behavior on residents’ health varies across three dimensions: urban versus rural areas, regions, and age groups.

#### 5.4.1 Urban–rural heterogeneity

The regression results based on urban–rural heterogeneity are presented in Table 5. The results show that the regression coefficient for reading behavior is significantly positive across both subsamples, with a larger absolute coefficient observed in the rural sample. This indicates that the positive association between reading behavior and physical and mental health is stronger among rural residents than among urban residents. From the perspective of resource construction, the likely reason is that urban areas, by leveraging their economic advantages, have concentrated high-quality medical resources and attracted experienced medical professionals. Thus, a relatively mature medical service system has been formed, enabling residents to access convenient professional treatment when facing health problems. Additionally, public cultural facilities centered on libraries, along with supporting reading services such as community reading rooms and newspaper kiosks, have become increasingly well-established, providing hardware support for residents to obtain health information. In contrast, rural areas are constrained by limited economic development, resulting in a weaker medical service system characterized by a shortage of medical professionals and insufficient service capacity, thereby making reading an important alternative pathway for rural residents to acquire health knowledge and improve their health.

**Table 5:** Heterogeneity analysis.

Variable	Urban–Rural		Region			Age group		
	Urban	Eastern	Eastern region	Central region	Western region	Young adults	Middle-aged adults	Older adults
Reading behavior (physical health)	0.0327^***^ (0.0049)	0.0540^***^ (0.0057)	0.0284^***^ (0.0064)	0.0508^***^ (0.0069)	0.0501^***^ (0.0061)	0.0247^***^ (0.0088)	0.0448^***^ (0.0053)	0.0362^***^ (0.0065)
Reading behaviors (mental health)	0.0162^***^ (0.0050)	0.0180^***^ (0.0058)	0.0160^***^ (0.0066)	0.0263^***^ (0.0070)	0.0217^***^ (0.0061)	−0.0385^***^ (0.0086)	0.0234^***^ (0.0055)	0.0343^***^ (0.0067)
Control variables	Yes	Yes	Yes	Yes	Yes	Yes	Yes	Yes
Year FE	Yes	Yes	Yes	Yes	Yes	Yes	Yes	Yes
Province FE	Yes	Yes	Yes	Yes	Yes	Yes	Yes	Yes
*N*	28,016	34,864	17,867	19,127	25,886	12,289	31,818	18,773

*, **, and *** indicate statistical significance at the 10, 5, and 1% levels, respectively.

From the perspective of service facilities, the possible reason is that urban and rural areas differ in the availability of health promotion channels, rendering reading a crucial means for rural residents to enhance their health. Urban areas possess a relatively well-developed health service system, including community fitness centers and sports parks equipped with diverse exercise apparatus, as well as offline health lectures and science popularization activities, all of which offer urban residents a variety of health promotion options. In contrast, rural residents face relatively limited health knowledge reserves and access channels, along with a lack of health-supporting facilities and low availability of exercise venues and health science popularization activities. Consequently, reading, being low-cost and easily accessible, becomes a common choice for rural residents.

#### 5.4.2 Regional heterogeneity

Given the imbalanced regional development in China, to more accurately capture regional heterogeneity, the sample is divided into three major regions—eastern, central, and western—based on the gradient of economic development. The regression results based on regional heterogeneity are presented in Table 5. The results show that the regression coefficient for reading behavior on physical health is significantly positive across the three regions, with a larger absolute coefficient observed in the central and western regions than in the eastern region. This suggests that reading behavior is more effective in promoting physical health for residents in the central and western regions than for those in the eastern region. The likely reason is that, in the economically developed eastern region, government investment in education is more substantial, and educational resources are not only of higher quality but also more equitably distributed. Consequently, residents in the eastern region are likely to possess stronger information discrimination skills and higher information literacy, leading to diminishing marginal returns in the improvement of health literacy through reading. In contrast, constrained by the uneven distribution and limited quality of educational resources, residents in the central and western regions tend to have relatively weak health literacy. Thus, reading serves as an effective means to bridge gaps in health literacy and promote health behaviors, thereby exerting a more pronounced positive effect on physical health.

Regarding mental health, reading behavior exerts a stronger positive effect in the central and western regions than in the eastern region. The possible reason is that the fast-paced urban life and high-intensity work pressure in the economically developed eastern region impose chronic stress on residents. This not only adversely affects mental health but also reduces the time and opportunities available for deep reading, resulting in reading behaviors that are predominantly fragmented and thus yielding limited mental health benefits. In contrast, the central and western regions are characterized by a slower pace of life, lower life pressure, and greater recreational opportunities. Such a lifestyle not only helps mitigate the occurrence of negative emotions at the source, thereby maintaining residents’ baseline mental health, but also provides ample discretionary time that facilitates conditions conducive to deep reading. Immersive reading allows readers to derive greater enjoyment from the reading experience, which in turn enhances reading pleasure. This process contributes to enriching mental well-being and alleviating psychological stress, thereby rendering the mental health benefits of reading more pronounced.

#### 5.4.3 Age heterogeneity

The regression results based on age heterogeneity are presented in Table 5. The results show that the regression coefficient for reading behavior on physical health is significantly positive for both groups, with a larger coefficient observed for the middle-aged and older adult group than for the younger group. This indicates that reading behavior is more effective in promoting physical health for the middle-aged and older adults than for their younger counterparts. The likely reason is that the younger group, being at a critical stage of life development, tends to allocate its time and energy to activities that yield direct returns, such as career development and social interaction, resulting in less time spent on health-related reading and a lower willingness to engage in it. In contrast, due to gradually declining physical functions and the consequent higher health risks, the middle-aged and older adult groups exhibits a significantly increased concern for health issues. Middle-aged individuals often present with suboptimal health status, whereas older adults tend to suffer from a range of chronic conditions. These health challenges render middle-aged and older populations more proactive in participating in reading activities, which not only contribute to the preservation of cognitive function and brain health, but also facilitate the acquisition of health-related knowledge and its subsequent translation into health-promoting practices and behaviors, thereby enhancing physical health outcomes. From the lens of cognitive aging, reading constitutes a low-threshold, home-based cultural pursuit that affords continuous cognitive stimulation and affective investment. Emerging evidence indicates that non-clinical, scalable, and home-delivered activities are beneficial for the maintenance of cognitive function in aging cohorts, and reading may exert its effects through analogous pathways, rendering it a readily disseminable strategy for cognitive preservation (Herold et al., 2024) (69).

Regarding mental health, the regression coefficients for both the middle-aged and older adult groups are positive, with the older adult group exhibiting a more pronounced effect. The likely reason is that the middle-aged group, which generally faces dual pressures from both workplace and family responsibilities, may find that reading helps them acquire knowledge and skills to meet the demands of social development, thereby alleviating psychological stress. For the older adult group, as they gradually withdraw from social production activities due to aging and declining physical functions and have yet to establish a correct self-perception of their new roles, reading provides positive emotional experiences, sustains their sense of self-worth, and helps them integrate into new social lifestyles, thereby effectively alleviating psychological anxiety and enhancing mental health. In contrast, for the younger group, reading behavior exhibits a significant negative effect on mental health. Research has found that the impact of sedentary behavior on health is also moderated by content attributes and context (34, 35). Therefore, the negative association between reading and health among adolescents may be attributed to the following reasons: in terms of reading medium, adolescents tend to prefer digital reading, which, although convenient, may diminish the emotional experience and emotion regulation afforded by deep reading, thereby affecting mental health; in terms of reading content, young adults may be more susceptible to the negative influence of adverse content, which may exacerbate the emergence of negative emotions; and in terms of reading duration and addictive behavior, some adolescents may develop reading addiction, which encroaches on time for studying and social activities, thereby adversely affecting their mental health.

## 6 Mechanism test

The regression results above show that higher reading frequency improves residents’ physical and mental health. Based on the theoretical analysis, this section further examines the mechanisms through which reading behavior affects physical and mental health by introducing three mechanism variables: cognitive ability, subjective class identity, and health risk perception bias. This paper adopts the approach of Jiang (59) and specifies the following model, as shown in Equation 2:

$$M_{\mathrm{it}}=\alpha +\text{βrea}d_{\mathrm{it}}+\gamma X_{\mathrm{it}}+\delta_{t}+\varphi_{p}+\varepsilon_{\mathrm{it}}$$ (2)

Where ( $M_{\mathrm{it}}$ ) represents the mechanism variables, including cognitive ability, subjective class identity, and health risk perception bias. The definitions of the remaining variables are the same as those in Equation 1.

### 6.1 Cognitive ability

The regression results in column (1) of Table 6 show that the coefficient of reading behavior is significantly positive, indicating that reading enhances residents’ cognitive ability, consequently contributing to better physical and mental health among them. The possible reasons are as follows. On the one hand, while reading literary works, readers often resonate with the emotions and situations of the characters, thereby enhancing their understanding of interpersonal relationships and social phenomena and improving cognitive ability. On the other hand, individuals with higher cognitive ability are not only better able to understand and apply health information, thereby exhibiting healthier behaviors (60), but also tend to choose and adhere to healthier lifestyles, including healthy habits (61) and reduced smoking.

**Table 6:** Mechanism test.

Variable	(1)	(2)	(3)
	Cognitive ability	Subjective class identity	Health risk Perception bias
Reading behavior	0.1370^***^ (0.0035)	0.0636^***^ (0.0036)	−0.0546^***^ (0.0195)
Control variables	Yes	Yes	Yes
Year FE	Yes	Yes	Yes
Province FE	Yes	Yes	Yes
*N*	62,880	62,880	2063

*, **, and *** indicate statistical significance at the 10, 5, and 1% levels, respectively.

### 6.2 Subjective class identity

The regression results in column (2) of Table 6 show that the coefficient of reading behavior is significantly positive, indicating that reading enhances residents’ subjective class identity, consequently contributing to better physical and mental health among them. This may be because reading enhances individuals’ decision-making ability through knowledge accumulation. For instance, rich financial knowledge and information help reduce household skill exclusion and optimize asset allocation (62). These decisions improve individuals’ socioeconomic status by enhancing their financial well-being, thereby strengthening their subjective class identity. Individuals with a higher subjective class identity tend not only to possess better abilities to cope with stress and regulate emotions (49), thereby reducing negative emotions and their adverse effects, but also to have greater health awareness and engage in healthier behaviors (53). These advantages further translate into daily life behaviors, helping individuals achieve better physical and mental health and improving their overall health status.

### 6.3 Health risk perception bias

The regression results in Column (3) of Table 6 show that the coefficient of reading behavior is significantly negative, indicating that reading reduces residents’ health risk perception bias, consequently contributing to better physical and mental health among them. The possible reasons are as follows. On the one hand, as a crucial channel for knowledge dissemination and information acquisition, reading enables individuals to directly acquire health-related knowledge, including the causes, prevention, and treatment of diseases. This reduces health risk perception bias arising from information asymmetry, thereby fostering accurate health judgments. Consequently, individuals are better equipped to make informed health decisions—such as purchasing health insurance (63)—which in turn promotes healthier behaviors and improves physical health. On the other hand, reading exposes individuals to diverse health-related cases, enabling them to observe and imitate healthy behaviors through vicarious learning. Moreover, successful case examples help alleviate anxiety by serving as positive role models, thereby reducing health risk perception bias. This facilitates a more accurate assessment of one’s health status, enhances self-confidence, and ultimately promotes mental well-being.

## 7 Conclusion and implications

Based on data from the China General Social Survey from 2012 to 2023, this study empirically examines the direct effects and underlying mechanisms of reading behavior on residents’ physical and mental health across the dimensions of physical health and mental health. The findings reveal that reading behavior significantly improves residents’ physical and mental health. The results of heterogeneity analysis indicate that the positive effect of reading behavior on residents’ health varies across urban–rural areas, regions, and age groups. In particular, the positive impact is stronger in rural areas and the central and western regions, where economic development is relatively lagging. Regarding age differences, the positive effect is stronger among middle-aged and older adult groups, whereas reading behavior exerts a significant negative effect on the mental health of the younger group. Mechanism analysis reveals that reading improves physical and mental health by enhancing cognitive ability, strengthening subjective class identity, and reducing health risk perception bias.

Based on the above conclusions, to fully leverage the health benefits of reading and promote the development of Healthy China, this paper offers the following policy implications:

Firstly, establish a cross-sectoral coordination mechanism integrating reading and health and strengthen the health benefits of reading. From the perspective of population public health, the core value of reading lies in three major characteristics: it is easy to implement, accessible to all, and scalable for widespread promotion. Existing research increasingly recognizes that low-intensity daily behaviors hold significant public health value (64). Reading, in this regard, is a typical low-barrier cultural activity that can be promoted through multiple channels, including libraries, communities, campuses, and online digital platforms.

Accordingly, public cultural institutions and health departments should deepen cooperation by integrating reading as an important auxiliary means into residents’ health management. Specifically, on the one hand, establish reading spaces in communities, hospitals, and other venues, and display books with positive messages—particularly health-related materials—to help readers develop accurate health perceptions. On the other hand, libraries can actively carry out reading promotion activities, including developing health reading lists and organizing “reading therapy” themed events, to fully leverage the therapeutic value of reading.

Secondly, implement differentiated reading provisions and precisely match the needs of different regions and population groups. The results show that rural residents, residents in the central and western regions, and middle-aged and older adult groups are the main beneficiaries, while the younger group may be susceptible to the negative influence of adverse content.

Behavioral health interventions cannot be detached from the reality of social inequality, as there are significant disparities in access to cultural, educational, and health resources across different regions and population groups (65). Therefore, reading promotion should not be implemented in a “one-size-fits-all” manner, but rather should adopt differentiated provision for different groups and regions. Research on geographically disadvantaged populations suggests that scalable and remotely deliverable interventions can help reduce barriers to resource access (66), implying that reading interventions should reach remote areas through extendable approaches such as digital reading resources and mobile library services. On this basis, increase investment in public cultural facilities—such as libraries and community reading rooms—in rural and central-western regions to narrow regional disparities and create more convenient reading opportunities. Furthermore, actively organize healthcare professionals and volunteers to carry out health knowledge popularization and offline lecture activities, thereby expanding residents’ access to health information and facilitating the efficient translation of reading-acquired knowledge into health behaviors.

In terms of differentiated services for population groups, for middle-aged and older adults, we should develop health-promoting and easy-to-understand reading materials focusing on cognitive maintenance, emotional regulation, and chronic disease management, so as to fully leverage the health benefits of reading as a cognitively active leisure activity. For young adults, we should provide positively oriented reading materials to help them avoid negative information, while also paying attention to their digital reading habits, sleep quality, physical exercise, and social participation, so as to prevent isolated reading from encroaching on time for other health-related behaviors, thereby promoting their mental health development.

Thirdly, continuously promote nationwide reading policies and expand the health benefits of reading. Existing research indicates that nationwide reading policies promote reading participation (15). Accordingly, continued policy support is required to further unleash the health value of reading. On the one hand, leverage the momentum of nationwide reading policies to continuously improve reading promotion activities, lower reading barriers, further stimulate reading interest among rural residents and middle-aged and older adult groups, increase reading participation rates, and further expand the health benefits of reading. On the other hand, libraries, as the main agents responsible for policy implementation, should optimize their collection structures according to the reading needs of different groups—especially by adding more health science books—so that collection resources can more accurately address residents’ health needs.

## 8 Limitations and future research directions

Using data from the Chinese General Social Survey (CGSS), this paper systematically analyzes the correlation, heterogeneity, and internal mechanisms between reading behavior and residents’ physical and mental health from two dimensions: physical health and mental health. Nevertheless, this study has several limitations that need to be addressed in follow-up research.

First, limitations regarding variable measurement indicators. Restricted by the CGSS dataset, variables such as reading behavior and health status rely on self-reported measurements, while relevant information including sleep conditions and digital media usage is unavailable, leading to omitted variables. In terms of research design, although propensity score matching (PSM) is adopted to mitigate selection bias arising from observable variables, the research design cannot rule out reverse causality and unobservable confounding factors.

Accordingly, future research may divide reading behavior into multi-dimensional indicators covering reading medium, reading content, reading duration, and other dimensions; adopt more objective health scales to capture objective health status; and employ panel data, instrumental variable approaches and other strategies to further identify causal relationships.

## Data Availability

The original contributions presented in the study are included in the article/supplementary material, further inquiries can be directed to the corresponding author.
